# 

**Published:** 1908-12

**Authors:** 


### The Library of the Bristol Medico-Chirurgical Society.

*The following donations have been received since the publication of the List in September.*

*November 30th*, 1908.

| | |
|---|---|
| J. W. Ballantyne, M.D. (1) .. .. .. .. | 1 volume. |
| J. Michell Clarke, M.D. (2) .. .. .. ... | 3 volumes. |
| F. R. Cross, M.B. (3) .. .. .. .. .. | 5 ,, |
| F. H. Edgeworth, M.D. (4) .. .. .. .. | 5 ,, |
| A. A. Lendon, M.D. (5) .. .. .. .. | 1 volume. |
| The Middlesex Hospital (6) .. .. .. .. | 2 volumes. |
| R. Shingleton Smith (7) .. .. .. .. | 5 ,, |
| W. Roger Williams (8) .. .. .. .. | 1 volume. |

### SEVENTIETH LIST OF BOOKS.

The titles of books mentioned in previous lists are not repeated.

The figures in brackets refer to the figures after the names of the donors, and show by whom the volumes were presented. The books to which no such figures are attached have either been bought from the Library Fund, or received through the *Journal*.

**Allbutt and H. D. Rolleston, Sir C.** [Eds.] *A System of Medicine.* Vol. IV. Pts. 1, 2 .. .. .. .. .. .. 1908

**Allen, R. W.** .. *Vaccine Therapy* .. .. .. .. 2nd Ed. 1908

**Baily, P. J.** .. .. *The Care and Nursing of the Insane* .. .. [1908]

**Ball, Sir C. B.** .. *The Rectum, its Diseases and Developmental Defects* .. .. .. .. .. .. .. .. .. .. 1908

**Ballantyne, J. W.** *The "Byrth of Mankynde:" its Author, Editions, and Contents* .. .. .. .. .. (1) 1907

**Ballet, G.** .. .. *Neurasthenia.* (Tr. from 3rd Edition by P. C. Smith) .. .. .. .. .. .. .. .. 1908

**Barnett, H. N.** .. *Legal Responsibility of the Drunkard* .. .. 1908

**Brown, W. L.** .. *Physiological Principles in Treatment* .. .. 1908

**Clark, D. N. Paton and G. H.** *Practical Course of General Physiology* 1908

**Cooper, A.** .. .. *The Sexual Disabilities of Man* .. .. .. 1908

**Dingwall-Fordyce, A.** *Diet in Infancy* .. .. .. .. .. .. .. 1908

**Emery, W. d'E.** .. *Clinical Bacteriology and Hæmatology* 3rd Ed. 1908

*Encyclopedia and Dictionary of Medicine and Surgery, Green's* Vol. IX. [1908]

**Erb, W.** .. .. *Handbook of Electro-Therapeutics.* (Tr. by L. Putzel) .. .. .. .. .. .. .. (7) 1883

**Goodall and J. W. Washbourn, E. W.** *A Manual of Infectious Diseases* .. .. .. .. .. .. .. .. 2nd Ed. 1908

**Gresswell, D. A.** .. *Natural History of Scarlatina* .. .. (7) 1890

**Groves, E. W. H.** *A Synopsis of Surgery* .. .. .. .. .. .. 1908

**Heine, B.** .. .. *Operations on the Ear.* (Tr. and Ed. from 2nd German Edition by W. L. Murphy) .. 1908

*Insane, Handbook for Attendants on the* .. .. .. .. 5th Ed. 1908

**James, J. B.** .. .. *Death and its Verification* .. .. .. .. 1908

**Kelynack, T. N.** [Ed.] *Tuberculosis in Infancy and Childhood* .. 1908

**Latimer, J.** .. .. *Sixteenth-Century Bristol* .. .. .. .. .. 1908

**Lendon, A. A.** .. *Nodal Fever* .. .. .. .. .. .. .. .. (5) 1905

**Luke, T. D.** .. .. *Natural Therapy* .. .. .. .. .. .. .. 1908

**MacIlwaine, S. W.** *The Future of Medicine* .. .. .. .. .. .. 1908

**Mackenzie, J.** .. *Diseases of the Heart* .. .. .. .. .. .. 1908

**Martindale and W. W. Westcott, W. H.** *The Extra Pharmacopœia* 13th Ed. 1908

**Mayou, M. S.** .. *Diseases of the Eye* .. .. .. .. .. .. .. 1908

**Morris, Sir M.** .. *Diseases of the Skin* .. .. .. .. [4th Ed.] 1908

**Murphy, D'A. Power and J. K.** [Eds.] *A System of Syphilis* Vol. II. 1908

**Oliver, T.** .. .. *Diseases of Occupations* .. .. .. .. .. [1907]

**Osler, W.** .. .. *Lectures on the Diagnosis of Abdominal Tumours* .. .. .. .. .. .. .. (7) 1894

**Parsons, J. H.** .. *The Pathology of the Eye.* Vols. I.—III. 1904-07

**Paton and G. H. Clark, D. N.** *Practical Course of General Physiology* 1908

**Power and J. K. Murphy, D'A.** [Eds.] *A System of Syphilis.* Vol. II. 1908
**Proctor, B. S.** .. *Manual of Pharmaceutical Testing* .. (4) 1890
**Probyn-Williams, R. J.** *A Practical Guide to the Administration of Anæsthetics* .. .. .. .. .. .. (4) 1901
*Quain's Elements of Anatomy.* Vol. III. .. .. .. 11th Ed. 1908
**Rawling, L. B.** .. *Landmarks and Surface Markings* 3rd Ed. 1908
**Rolleston, Sir C. Allbutt and H. D.** [Eds.] *A System of Medicine* Vol. IV. Pts. 1, 2 .. .. .. .. .. .. 1908
**Russell, R.** .. .. *Strength and Diet* .. .. .. .. .. .. (7) 1905
**Sutherland, G. A.** [Ed.] *A System of Diet and Dietetics* .. .. .. .. 1908
**Swayne, J. G.** .. *Obstetric Aphorisms* .. .. .. (4) 8th Ed. 1884
**Tredgold, A. F.** .. *Mental Deficiency* .. .. .. .. .. .. .. 1908
**Vale, F. P.** .. .. *Has Surgical Treatment lessened the Mortality from Appendicitis ?* .. .. .. .. .. .. 1908
**Walker, J. W. T.** .. *Estimation of the Renal Function in Urinary Surgery* .. .. .. .. .. .. .. .. .. .. 1908
**Washbourn, E. W. Goodall and J. W.** *A Manual of Infectious Diseases* .. .. .. .. .. .. .. 2nd Ed. 1908
**Westcott, W. H. Martindale and W. W.** *The Extra Pharmacopœia* 13th Ed. 1908
**White, W. H.** .. *Common Affections of the Liver* .. .. .. 1908
**Wiggins, W. D.** .. *Midwifery for Midwives* .. .. .. .. .. .. 1904
**Williams, W. R.** .. *The Natural History of Cancer* .. .. (8) 1908

### TRANSACTIONS, REPORTS, JOURNALS, &c.

American Journal of Obstetrics, The .. .. .. .. .. Vol. LVII. 1908
American Laryngological Association, Transactions of the .. .. 1908
Annales d'Oculistique .. .. .. .. .. (3) Tom. XCIII., XCIV. 1885
Archives d'Ophtalmologie .. .. .. (3) Tom. IX.—XII. 1889–92
Archives of the Middlesex Hospital .. (6) Vols. XII., XIII. 1908
Boston Medical and Surgical Journal, The .. .. .. Vol. CLVIII. 1908
Brain .. .. .. .. .. .. .. .. .. .. (4) Vols. IX., 1887, XI. 1889
British Journal of Tuberculosis, The .. .. .. .. .. Vol. I. 1907
Echo médical du Nord, L' .. .. .. .. .. .. .. .. .. .. .. .. 1906
Edinburgh Obstetrical Society, Transactions of the Vols. XXXI.—XXXIII. 1906-08
Hospital, The .. .. .. .. .. .. .. .. .. .. .. Vol. XLIII. 1908
Journal of Medical Research, The .. .. .. .. .. Vol. XVIII. 1908
Journal of Obstetrics and Gynæcology, The .. .. .. Vol. XIII. 1908
Journal of Physiology, The .. .. .. (2) Vols. XVI.—XVIII. 1894-95
Journal of the American Medical Association, The .. Vol. L. 1908
Library World, The .. .. .. .. .. .. .. .. .. Vol. X. 1908
"Medical Review," Index to Vols. I.—X. .. .. .. .. .. .. .. 1908
Philippine Journal of Science, The .. .. .. .. .. .. Vol. II. 1907
Progressive Medicine .. .. .. .. .. .. .. .. .. .. Vol. III. 1908
Revue hebdomadaire de Laryngologie .. Tom. XXVIII., Vol. I. 1908
Royal Society of Medicine, Proceedings of the .. Vol. I., 3 parts 1908
Univ. of Penna. Medical Bulletin .. .. .. .. .. .. Vol. XX. 1908

Prevention of Disease. The Indian Government had in contemplation to appoint Lieutenant Knight when he would have attained his captaincy, a few months hence, to do special sanitary work in the Bombay Presidency.—*Medical Press and Circular.*

The fellow-students and friends of Dr. Knight in Bristol will receive the above information with much regret.

### Local Medical Notes.

### BRISTOL.

**University College.**—The following examinations have recently been passed :—

F.R.C.S. Edin.—J. H. Board, W. W. King.

Conjoint Board.—*Biology:* R. I. Dacre. *Chemistry:* C. Kingston. *Physics:* R. I. Dacre, C. Kingston, A. G. Morris. *Pharmacy:* W. Worger, B. G. Derry, H. H. Templeton. *Anatomy and Physiology:* T. E. Ashley. *Surgery:* E. E. Davies,* E. R. Sircom.*

L.D.S. Eng.—*Chemistry and Physics:* P. L. Ealand, B. L. Morgan, C. V. Osborne.

R.A.M.C.—G. S. Parkinson, M.R.C.S.Eng.

Captain F. Percival Mackie, F.R.C.S., of the Indian Medical Service, and assistant in the Bombay Bacteriological Laboratory, has been selected by the Government of India to join Sir David Bruce's expedition, which is on the way to Uganda to investigate further the disease known as "sleeping sickness." Dr. Mackie was formerly House Surgeon at the Eye Hospital, Bristol, also Assistant House Physician at the Bristol General Hospital, and Medical Superintendent at the Ham Green City Hospital.

**Bristol Royal Infirmary.**—The following appointments have been recently made :—*House Surgeon and Senior Resident Officer:* Hubert Chitty, M.S.Lond., F.R.C.S. *Casualty Officer:* E. W. Squire, M.B., B.S.Lond. *Assistant Anæsthetist:* Leonard A. Moore, M.R.C.S., L.R.C.P.Lond. *Medical Registrar:* Cyril Claude Lavington, M.B., B.S.Durh. *Dental Anæsthetist:* J. F. Blackett, M.B., B.S.Lond.

**Bristol General Hospital.**—The following appointments have been recently made :—*Honorary Skiagraphist:* John Ellington Jones, L.R.C.P.Lond., M.R.C.S., L.S.A. *Assistant Skiagraphist:* Frank Gower Bergin, L.R.C.P.Lond., M.R.C.S.

* Denotes qualification.

The half-yearly meeting of the General Board of this Hospital was held on Monday, September 14th, Mr. J. Storrs Fry being in the chair. The Chairman in his remarks said that the position of the institution was, on the whole, not so satisfactory as could be wished, for there was a considerable falling off in the receipts, while the expenditure had also increased, arising from the increased cost of provisions and a greater expenditure in coal and medical and surgical sundries. There had been an increase in the number of out-patients, and this had entailed increased cost. The addition to the nurses' home was now complete and in occupation, and was proving of great value. An addition had been made to the dental department, and probably further rooms would be required soon. Mr. Hare gave further details of the increased cost, and said that the total excess of expenditure over last year was close on £670, and the decrease in the receipts was £900, so that they were behind last year by a large sum. In the out-patient department there had been an increase of 2,158, the total being the large number of 19,059, but he did not know whether this was due to the distress among the working classes. An alteration in the rules was made, adding a skiagraphist to the Staff.

**Bristol Dispensary.**—James Francis Blackett, M.B., B.S.Lond., and H. Finzel, M.D.Lond., have been appointed Medical Officers.

**Cossham Memorial Hospital.**—E. V. Connellan, M.R.C.S., L.R.C.P., has been appointed House Surgeon.

**Children's Hospital.**—J. A. Brydon, M.B., Ch.B.Edin., has been appointed Junior House Surgeon.

**Müller's Orphanage Homes.**—Elliott Thornton Glenny, M.B., B.S.Lond., L.R.C.P., M.R.C.S., has been appointed Medical Officer.

**Queen Victoria Jubilee Convalescent Home, Durdham Downs.**—A bronze tablet has recently been placed in the entrance lobby of this institution. It bears the following inscription :—

> "In honour of Her Gracious Majesty Queen Victoria; in memory of a Beloved Wife; and in sympathy with the suffering, this house was given by Sir E. P. Wills, for a Convalescent Home, on the occasion of the 60th Anniversary of Her Majesty's Accession, June 20th, 1897, and the Home was opened by Her Majesty, on November 15th, 1899. A.D. 1908."

This Convalescent Home is doing excellent work, and since its opening 12,400 patients have been admitted.

**The late Dr. Emily E. Eberle, of Clifton.**—As a memorial to the late Dr. Emily Eberle, a free bed bearing her name is to be placed at the Private Hospital for Women, Berkeley Square, Clifton.

**Royal Territorial Medical Service.**—The *London Gazette* of September 29th contained the list of *à la suite* officers of the various Territorial Hospitals. The following list will be of interest in this district:—

SECOND SOUTHERN GENERAL HOSPITAL.—The following to be officers whose services will be available on mobilisation, Sept. 30th, 1908: To be Lieutenant-Colonel—R. S. Smith, M.D., E. C. Board, J. M. Clarke, M.D., and G. M. Smith. To be Majors—A. B. Prowse, M.D., F.R.C.S.Eng., J. Swain, M.D., F.R.C.S.Eng., G. Parker, M.D., C. A. Morton, F.R.C.S.Eng., P. W. Williams, M.D., R. G. P. Lansdown, M.D., C. H. Walker, M.B., F.R.C.S.Eng., and W. R. Ackland. To be Captains—F. H. Edgeworth, M.D., J. L. Firth, M.D., F.R.C.S.Eng., J. O. Symes, M.D., T. Carwardine, M.B., F.R.C.S.Eng., J. Taylor, H. G. Kyle, M.B., C. F. Coombs, M.D., H. F. Mole, F.R.C.S.Eng., E. C. Williams, M.B., E. W. H. Groves, M.D., F.R.C.S.Eng., J. M. Fortescue-Brickdale, M.D., E. H. E. Stack, M.B., F.R.C.S.Eng., J. Freeman, M.D., F.R.C.S.Edin., J. Dacre, J. J. S. Lucas, M.D., H. E. Harris, M.B., F.R.C.S.Eng., W. Cotton, M.D., H. Hill, M.D., and W. S. V. Stock, M.B.

Recruits, who will be trained in hospital duties, are wanted for a General Hospital in Bristol. Men who take an interest in military matters, and especially in ward work and hospital nursing, are invited to take this opportunity of doing good service for their country by joining. They must be between the ages of 17 and 35.

J. PAUL BUSH, C.M.G., *Administrator.*

### BATH.

**Bath Eye Infirmary.**—E. Daubney Hancock, M.R.C.S., L.R.C.P., has been appointed Honorary Surgeon.

**The Winsley Sanatorium for Consumptive Patients.**—The quarterly meeting of the Board of Governors of the Winsley Sanatorium was held at Bristol on September 16th, under the presidency of Dr. R. Shingleton Smith. It was decided to admit patients from outside the counties of Gloucester, Wilts and Somerset on payment of two guineas weekly, so long as beds were available without depriving patients within the three counties. The medical officer of the institution having resigned, it was decided to advertise the appointment, the salary to be £200 per annum, rising by £20 a year to £300. The financial position of the Sanatorium was carefully considered, and it was stated that an additional income of £600 a year was required.

Leonard Crossley, M.D., Ch.B.Edin., has been appointed Resident Medical Officer.

### CARDIFF.

**The Cardiff Infirmary.**—Colonel Bruce Vaughan, Chairman of the House Committee of the Cardiff Infirmary, announced at the meeting held on September 16th, that an anonymous gift of £10,000 had been received from a Glamorganshire coal owner towards the new wing. An effort is being made to raise an additional income of £7,000 per annum, and last May Mr. J. Cory promised to add 25 per cent. to all sums received. In response a Welsh colliery owner sent £1,000, Mrs. J. Nixon sent 10,000 guineas, Mr. H. Webb subscribed £3,000, and another £1,000 was contributed anonymously. The latest gift brings the total to £25,700.

### DEVON.

**The Health of North Devon in 1907.**—Dr. E. J. Slade-King in his summary of the reports of the medical officers of health of North Devon for 1907, which he has just presented to the Devon County Council, states that on an estimated population of 146,775 the death-rate was 14.3, compared with 13.7 in 1906. The annual death-rate per 1,000 births under one year of age was 104; in 1906 it was 98.7. Ninety-six deaths occurred from zymotic diseases, giving a death-rate of 0.7 per 1,000. Six deaths were due to enteric fever. 467 cases of infectious disease were notified, against 430 in 1906. 101 deaths were due to pulmonary tuberculosis, equal to a death-rate of 0.6 per 1,000; in 1901 there were 150 deaths from this disease, and in 1906 the number was 123. 121 deaths were caused by cancer, compared with 123 in 1906. The birth-rate was 19.7 per 1,000, as against 21 per 1,000 in 1906.

**Hospital Saturday at Newton Abbot.**—£274 were collected recently in Newton Abbot for the Hospital Saturday Fund. This is £54 in excess of any previous Hospital Saturday collection.

**Hospital Sunday at Exeter.**—October 18th, St. Luke's Day, was celebrated as Hospital Sunday at Exeter Cathedral, when collections were made for the Royal Devon and Exeter Hospital. The Committee, Staff and Governors of that institution met the mayor and other representatives of civic bodies at the Guildhall and walked in procession to the cathedral to attend the afternoon service.

### LYDNEY.

**New Hospital for Lydney (Gloucestershire) and District.**—On September 24th, Sir Charles Dilke, M.P., in the presence of a large gathering, formally opened the new hospital for Lydney and district. The building comprises two wards of four beds each, operation theatre, dispensary, &c. The site was given by Mr. C. Bathurst, and Mr. R. Thomas has defrayed the whole cost of building the hospital. Mr. Bathurst suggested as a means of providing an "Endowment Fund," that if the public would subscribe £800 for this purpose, by the time the hospital was completed, he would make the sum up to £1,000. The public has responded to the appeal, and there is no doubt that the required amount will be raised.

### TAUNTON.

Over £100 were raised as a result of the Carnival recently held at Taunton. After payment of expenses, £5 of this will be given to the Bristol Eye Infirmary, and the remainder will be divided between the Taunton and Somerset Hospital and the Taunton and District Nursing Association.

### WESTON-SUPER-MARE.

**Weston-super-Mare Hospital.**—The annual house-to-house collection by members of the local friendly societies, which was recently held at Weston-super-Mare, resulted in £52 being raised for the funds of the Weston-super-Mare Hospital.

---

**National Association for the Feeble Minded : Bristol Meeting.**—Conferences of the After-Care Committees and of the National Special Schools Union.—The judicious care of the feeble-minded is of such far-reaching and national importance, that the recent Conference in Bristol on the subject merits the earnest attention of all medical practitioners. The Conference was largely attended, and included several of our profession from a distance, together with many others engaged in the practical work of the societies concerned.

Public opinion in reference to the treatment of the mentally defective is at present ill-informed. There are people who recommend the sterilisation of these children in early life, as the only adequate means of preventing the perpetuation of the evil. But as we do not seem to be within measurable distance of this proceeding, it will be well for us to determine what is the best possible thing in the immediate future.

The necessity of detaining, under proper control, persons suffering from lunacy of a nature which renders them dangerous to themselves or to the community is universally recognised. Those other forms of mental aberration in which the danger, although not so obvious, is none the less real, should be dealt with by legislation, and the most hopeful results can only be attained by detention in suitable residential institutions.

The good results of work in this direction are admirably set forth in the Ninth Annual Report, 1907, of The Incorporated Lancashire and Cheshire Society for the Permanent Care of the Feeble-Minded, of which Miss Mary Dendy, of 13 Clarence Road,

Withington, Manchester, is the honorary secretary. Voluntary effort such as this is, however, hardly to be expected in less wealthy districts, in which practical difficulties, well-nigh insuperable, would present themselves. But the report mentioned is worth the careful reading of anyone interested in the subject.

The members of the Conference were welcomed on Thursday, October 22nd, 1908, by the Chairman of the Bristol Education Committee, Dr. E. H. Cook, who called attention to the great importance of the matters that would come before them, emphasising the need not only of suitable instruction for the physically and mentally defective during school life, but also the necessity of placing the children in the time immediately afterwards in homes where they could be properly trained. After-care committees had been formed in several of the large towns, and Bristol had been active in the voluntary work of doing the best for these children under existing circumstances.

Sir William Chance, the Chairman of the National Association, gave statistics of the work done by the After-Care Committees, and alluded to the recent Report of the Royal Commission, which should be exceedingly useful to these voluntary committees, and should give them much encouragement in their efforts.

On the same day the following papers were read and discussed:—

"The Permanent Care of Defectives." Mr. J. S. Legge, Director of Education at Liverpool, and late H.M. Inspector of Industrial and Reformatory Schools.

"The Care of Defectives in Workhouses." Mr. J. J. Simpson, Clerk to the Bristol Board of Guardians.

"The Detection and Treatment of the Feeble-Minded." Dr. W. R. Dawson, late Examiner in Mental Diseases, Dublin University.

"The Care and Treatment of Epileptics." Dr. W. A. Turner, Chalfont Colony, St. Peter's, Buckinghamshire.

In the evening Dr. Frederic Rose, Assistant Educational Adviser to the London County Council, gave a lantern lecture on "Open-air Schools." These were first established in Germany. The London County Council have three such schools, and Bradford has one. It is claimed that under this arrangement the children show physical, educational and moral improvement in a marked degree.

On Friday morning members assembled in large numbers at Redcross Street School to inspect the physically and mentally defective classes which are held there, and then returned to the other work of the Conference, when Mr. C. E. H. Hobhouse, M.P., who is the President of the National Special Schools Union, and was a member of the Royal Commission, gave an address on the Report, which he said revealed the gravity and the complexity of the problem which confronted them, and that although there were certain voluntary agencies looking after some of these unfortunate beings at the critical period of their leaving school, yet the greater portion of them were entirely unhelped and uncontrolled. The permissive Act of 1899 had not fulfilled expectations. One serious defect in it is that it brings the training to an end just at the most dangerous period of the defective child. At no distant date the problem of how to deal adequately with these young people must be grappled with, and it seemed clear that there would have to be but one authority, which at all stages of life, and for all purposes of training or of education, should be responsible for all restraint, and should be able to provide for all development, so that no longer should these unfortunate people be at large in the world, a danger and a nuisance to themselves, a burden and a grievance to the community, and a source of unnecessary anxiety and expense.

The papers for the day were :—

"The Outlook for Special Schools in London." Mrs. Burgwin, London.

"The Special Value of Residential Institutions in the Training of Defective Children." Mr. C. H. Wyatt, Director of Elementary Education, Manchester.

"The Classification of Mental Deficiency in Relation to School Work." Dr. T. M. Carter, Bristol.

"Methods of Teaching Defective Children." Miss Brookes, Wolverhampton Special School.

On Saturday the papers were :—

"The Result of Deafness on Education." Dr. T. A. Green, Bristol.

"Schools for Physically Defective Children." Miss Collard, Assistant-Superintendent of Physically Defective School, London County Council.

"The Training of Epileptic Children." Mr. A. E. Lewis, Head Master of Lingfield School for Epileptic Children.

Naturally the greater consideration of the Conference was given to the mentally defective, as it is with them the principal difficulties lie. But the physically defective require most careful training in order to enable them to do something substantial, if not everything, towards supporting themselves when they leave school. The discussions were well maintained, and the whole proceedings were excellently reported in the local papers.

The social side of the occasion was well managed. On Thursday evening the representatives were entertained by the Local Committee at University College, and on Friday evening the Lord Mayor gave an admirable reception in our well-equipped Museum and our attractive Art Gallery. The Local Committee, in whose hands the arrangements were, was a representative one, with Dr. T. M. Carter as its Chairman, Mr. Clark its Treasurer, and Miss Townsend and Miss Waring Adams its Secretaries.

### INDEX.

Alexander, Dr. D. A.—Abdominal fibroma simulating splenomegaly, 60.
Aneurism, The plastic operations for, 68.
Arterial suture, 65.
Arterio-sclerosis, 335.
Arteriotomy for embolus and thrombosis, 67.
Aural complications of influenza and their treatment—H. F. Mole, 226.

Basal hemorrhage accompanying dilatation of posterior cerebral vessels—Dr. T. M. Carter, 322.
Basic meningitis with temporary amaurosis, Case of, 373.
Bladder, Radical treatment of carcinoma of, 339.
Blood-vessels, Rheumatism and disease of the, 245.
Blood-vessels, Surgery of the, 65.
Books, Reviews of—
- Adamson, Dr. H. G.—Skin affections in childhood, 277.
- Alcohol and the human body—Sir V. Horsley and Mary D. Sturge, 80.
- Allbutt, Drs. W. S. Playfair and T. W. Eden, Sir T. C. [Eds.]—A system of gynæcology, 264.
- Allbutt and Dr. H. D. Rolleston, Sir T. C. [Eds.]—A system of medicine, 69, 349.
- Allen, Dr. R. W.—The opsonic method of treatment, 277.
- American Dermatological Association, Transactions of the, 81.
- Anatomical terminology—Dr. L. F. Barker, 275.
- Anatomy, Manual of practical—Dr. D. J. Cunningham, 170.
- Anatomy, Surgical applied—Sir F. Treves, 170.
- Anatomy, The pocket—C. H. Fagge, 171.
- Anus, Diseases of the rectum and—H. Cripps, 269.

Books, Reviews of (*continued*)—
- Arterial hypertonus, sclerosis and blood pressure—Dr. W. Russell, 254.
- Atkinson, Dr. S. B.—The law in general practice, 349.
- Bacteriology, Manual of—Drs. R. Muir and J. Ritchie, 353.
- Bardswell and J. E. Chapman, Drs. N. D.—Diets in tuberculosis, 353.
- Barker, Dr. L. F.—Anatomical terminology, 275.
- Barton, Dr. G. A. H.—A guide to the administration of ethyl chloride, 278.
- Barwell, Dr. H.—Diseases of the larynx, 356
- Bennie, Dr. P. B.—Rational and effective treatment of hip-disease, 268.
- Bilharziosis—Dr. F. C. Madden, 82.
- Blutes, Atlas der klinischen mikroscopie des—Drs. E. Meyer und H. Rieder, 343.
- Brockbank, Dr. E. M.—Life assurance and general practice, 352.
- Calmette, A.—Les venins des animaux venimeuse et la serothérapie antivenimeuse, 276.
- Campbell, Dr. H.—Aids to pathology, 353.
- Campbell, Dr. J.—A text-book of treatment: obstetrics and gynecology, 268.
- Cancer—S. and G. E. Keith, 347.
- Cancer of the rectum—H. Cripps, 269.
- Cancer of the stomach—A. W. M. Robson, 165.
- Cancer, Stray leaves and some fruit on—Dr. H. D. McCulloch, 354.
- Cancer, The reduction of—Hon. R. Russell, 276.
- Catalogue (Index-) of the library of the Surgeon-General's office, 352.

Books, Reviews of (*continued*)—
Cerebellum, Tumours of the—Dr. J. Wyllie, 262.
Childhood, Skin affections in—Dr. H. G. Adamson, 277
Childhood, Tuberculosis in—Dr. T. N. Kelynack [Ed.], 350.
Children, The treatment of disease in—Dr. G. A. Sutherland, 169.
Clarke, Dr. J. J.—Protozoa and disease, 354.
Clubbe, C. P. B.—The diagnosis and treatment of intussusception, 272.
Consumption, home treatment and rules for living—Dr. H. W. Crowe, 169.
Corner and H. I. Pinches, Drs. E. M.—Operations of general practice, 271.
Cripps, H.—Cancer of the rectum, 269; On diseases of the rectum and anus, 269.
Crowe, Dr. H. W.—Consumption, home treatment and rules for living, 169.
Cunning, J.—Aids to surgery, 274.
Cunningham, Dr. D. J.—Manual of practical anatomy, 170.
Dampier-Bennett, A. G.—Physical methods in the treatment of heart disease, 356.
Deanesly, Dr. E.—Modern methods of diagnosis in urinary surgery, 272.
Degeneration in families—Dr. F. Lange, 78.
Diets in tuberculosis—Drs. N. D. Bardswell and J. E. Chapman, 353.
Diphtheria in practice—Dr. W. F. Litchfield, 258.
Disease, its cause and prevention—Dr. G. E. Richmond, 353.
Disinfectants, The bacteriological examination of—W. Partridge, 276.
Dore, Sir M. Morris and S. E.—Light and X-ray treatment of skin diseases, 351.
Eden, Sir T. C. Allbutt, Drs. W. S. Playfair and T. W. [Eds.]—A system of gynæcology, 264.
Encyclopædia and dictionary of medicine and surgery, Green's, 256.

Books, Reviews of (*continued*)—
Endometrium, A contribution to the pathology of the—Dr. Jessie M. Macgregor, 167.
Epilepsy—Dr. W. A. Turner, 261.
Esmarch, Dr. F.—First aid to the injured, 273.
Ethyl chloride, A guide to the administration of—Dr. G. A. H. Barton, 278.
Eye, Diseases of the—Dr. J. H. Parsons, 354.
Fagge, C. H.—The pocket anatomy, 171.
Feindel, H. Meige and E.—Tics and their treatment, 262.
Fernie, Dr. W. T.—Precious stones, 348.
Fevers in the tropics—Dr. L. Rogers, 257.
First aid to the injured—Dr. F. Esmarch, 273; Drs. F. J. Warwick and A. C. Tunstall, 274.
Fortescue-Brickdale, Drs. F. Francis and J. M.—The chemical basis of pharmacology, 72.
Francis and J. M. Fortescue-Brickdale, Drs. F.—The chemical basis of pharmacology, 72.
Goodall, Drs. G. H. Savage and E.—Insanity and allied neuroses, 264.
Groves, E. W. H.—A synopsis of surgery, 356.
Gynæcology, A system of—Sir T. C. Allbutt, Drs. W. S. Playfair and T. W. Eden [Eds.], 264.
Gynecology and obstetrics—Dr. J. Campbell, 268.
Hare, Dr. H. A.—A text-book of the practice of medicine, 164.
Hart, Dr. A. H.—Some successful prescriptions, 259.
Heart disease and thoracic aneurysm—Dr. F. J. Poynton, 253.
Heart disease, Treatment by physical methods—A. G. Dampier-Bennett, 356.
Hebblethwaite, Dr.—Chart, 352.
Henry Phipps Institute for tuberculosis, Third annual report of the, 259.
Hernia, its cause and treatment—R. W. Murray, 275.

Books, Reviews of (*continued*)—
Hip-disease, Rational and effective treatment of—Dr. P. B. Bennie, 268.
History, A chronology of medical —Dr. J. Young, 256.
Horsley and Mary D. Sturge, Sir V.—Alcohol and the human body, 80.
Hutchison and H. S. Collier, Drs. R. [Eds.]—An index of treatment, 343.
Hygiene and public health—Drs. L. C. Parkes and H. R. Kenwood, 355.
Hysteria, The major symptoms of—Dr. P. Janet, 261.
Infancy, Tuberculosis in—Dr. T. N. Kelynack [Ed.] 350.
Infectious diseases, The prevention of—Dr. J. C. M'Vail, 259.
Insanity and allied neuroses—Drs. G. H. Savage and E. Goodall, 264.
Insanity, its causes and prevention—C. Williams, 263.
Internal secretions and the principles of treatment, The—Dr. C. E. de M. Sajous, 166.
Intussusception, Diagnosis and treatment of—C. P. B. Clubbe, 272.
Janet, Dr. P.—The major symptoms of hysteria, 261.
Johns Hopkins Hospital reports, 273.
Jones, Dr. R.—A text-book of mental and sick nursing, 278.
Keith, S. and G. E.—Cancer: relief of pain and possible cure, 347.
Kelynack, Dr. T. N. [Ed.]—Tuberculosis in infancy and childhood, 350.
Kenwood, Drs. L. C. Parkes and H. R.—Hygiene and public health, 355.
Lange, Dr. F.—Degeneration in families, 78.
Larynx, Diseases of the—Dr. H. Barwell, 356.
Laveran, A.—Traitê du paludisme, 258.
Law in general practice—Dr. S. B. Atkinson, 349.
Life assurance in general practice —Dr. E. M. Brockbank, 352.
Life, The prolongation of—E. Metchnikoff, 74.

Books, Reviews of (*continued*)—
Light and X-ray treatment of skin diseases—Sir M. Morris and S. E. Dore, 351.
Litchfield, Dr. W. F.—Diphtheria in practice, 258.
Lung, Hygiene of the—Dr. L. v. Schrötter, 171.
Macfie, Dr. R. C.—The romance of medicine, 172.
Macgregor, Dr. Jessie M.—A contribution to the pathology of the endometrium, 167.
M'Vail, Dr. J. C.—The prevention of infectious diseases, 259.
Madden, Dr. F. C.—Bilharziosis, 82.
Maisonneuve, I.—The experimental prophylaxis of syphilis, 355.
Manson, Sir P.—Tropical diseases, 171.
McCulloch, Dr. H. D.—Stray leaves and some fruit on cancer and tuberculosis, 354.
Medical annual, The, 80.
Medicine, Minor—Dr. W. E. Wynter, 169.
Medicine, System of—Sir T. C. Allbutt and Dr. H. D. Rolleston [Eds.], 69, 349.
Medicine, Text-book of the practice of—Dr. H. A. Hare, 164.
Medicine, The romance of—Dr. R. C. Macfie, 172.
Meige and E. Feindel, H.—Tics and their treatment, 262.
Mental and sick nursing, Text-book of—Dr. R. Jones, 278.
Metchnikoff, E.—The prolongation of life, 74.
Meyer und H. Rieder, Drs. E.—Atlas der klinischen mikroscopie des blutes, 343.
Micholitsch, E. Wertheim and T. —The technique of vaginoperitoneal operations, 267.
Midwifery for nurses and midwives, Rotunda—Dr. G. T. Wrench, 268.
Midwifery, Rotunda practical—Drs. E. H. Tweedy and G. T. Wrench, 268.
Miles and A. Thomson, A.—Manual of surgery, 269.
Morris and S. E. Dore, Sir M.—Light and X-Ray treatment of skin diseases, 351.
Muir and J. Ritchie, Drs. R.—Manual of bacteriology, 353.

Books, Reviews of (*continued*)—
Murphy, Drs. D'A. Power and J. K. [Eds.]—A system of syphilis, 344.
Murray, R. W.—Hernia, its cause and treatment, 275.
Murrell, Dr. W.—What to do in cases of poisoning, 351.
Nerves, Injuries of—J. Sherren, 263.
Neurasthenia, Clinical lectures on —Dr. T. D. Savill, 80.
Neurology, Department of, Harvard Medical School, Collected papers, 82, 263.
New York, History of the Medical Society of the State of, 257.
New York post-graduate Medical School and Hospital, Contributions to medicine and surgery, 257.
Obstetrics and gynecology—Dr. J. Campbell, 268.
Operations of general practice—Drs. E. M. Corner and H. I. Pinches, 271.
Opsonic method of treatment, The—Dr. R. W. Allen, 277.
Paludisme, Traité du—Dr. A. Laveran, 258.
Parkes and H. R. Kenwood, Drs. L. C.—Hygiene and public health, 355.
Parsons, Dr. J. H.—Diseases of the eye, 354.
Partridge, W.—The bacteriological examination of disinfectants, 276.
Pathology, Aids to — Dr. H. Campbell, 353.
Pharmacology, The chemical basis of—Drs. F. Francis and J. M. Fortescue-Brickdale, 72.
Philadelphia, Transactions of the College of Physicians of, 258.
Photographic exposure record and diary, 352.
Pinches, Drs. E. M. Corner and H. I. — The operations of general practice, 271.
Playfair and T. W. Eden, Sir T. C. Allbutt, Drs. W. S. [Eds.]—A system of gynæcology, 264.
Pneumonia, Acute—Dr. S. Taylor, 167.
Poisoning, What to do in cases of —Dr. W. Murrell, 351.
Post-graduate clinical studies—Dr. H. H. Scott, 82.

Books Reviews of (*continued*)—
Power and J. K. Murphy, Drs. D'A. [Eds.] — A system of syphilis, 344.
Poynton, Dr. F. J.—Heart disease and thoracic aneurysm, 253.
Precious stones — Dr. W. T. Fernie, 348.
Prescriptions, Some successful—Dr. A. H. Hart, 259.
Prostate, Hypertrophy and cancer of the, 273.
Protozoa and disease—Dr. J. J. Clarke, 354.
Quarterly journal of medicine, The, 82.
Rectum and anus, On diseases of the—H. Cripps, 269.
Rectum, Cancer of the—H. Cripps, 269.
Rectum, Surgery of the—F. C. Wallis, 270.
Richmond, Dr. G. E.—Disease, its cause and prevention, 353.
Ritchie, Drs. R. Muir and J. — Manual of bacteriology, 353.
Robson, A. W. M.—Cancer of the stomach, 165.
Rogers, Dr. L.—Fevers in the tropics, 257.
Rolleston, Sir T. C. Allbutt and Dr. H. D. [Eds.]—A system of medicine, 69, 349.
Royal society of medicine, The proceedings of the, 172.
Russell, Hon. R.—The reduction of cancer, 276.
Russell, Dr. W.—Arterial hypertonus, sclerosis, and blood pressure, 254.
Sajous, Dr. C. E. de M.—The internal secretions and the principles of medicine, 166.
Sargent, Dr. P.—Surgical emergencies, 271.
Savage and E. Goodall, Drs. G. H.—Insanity and allied neuroses, 264.
Savill, Dr. T. D.—Clinical lectures on neurasthenia, 80.
Schrötter, Dr. L. v.—Hygiene of the lung in health and disease, 171.
Scott, Dr. H. H.—Post-graduate clinical studies, 82.
Sherren, J.—Injuries of nerves and their treatment, 263.
Skin affections in childhood—Dr. H. G. Adamson, 277.

Books, Reviews of (*continued*)—
Skin diseases, Light and X-ray treatment of—Sir M. Morris and S. E. Dore, 351.
Spinal cord, Diseases of the—Dr. R. T. Williamson, 259.
Stomach, Cancer of the—A. W. M. Robson, 165.
Sturge, Sir V. Horsley and Mary D.—Alcohol and the human body, 80.
Surgery, A synopsis of—E. W. H. Groves, 356.
Surgery, Aids to—J. Cunning, 274.
Surgery, Manual of—A. Thomson and A. Miles, 269.
Surgical emergencies — Dr. P. Sargent, 271.
Sutherland, Dr. G.—The treatment of disease in children, 169.
Syphilis, A system of—Drs. D'A. Power and J. K. Murphy [Eds.], 344.
Syphilis, The experimental prophylaxis of—P. Maisonneuve, 355.
Taylor, Dr. S.—Acute pneumonia, 167.
Thomson and A. Miles, A.—Manual of surgery, 269.
Tics and their treatment—H. Meige and E. Feindel, 262.
Treatment, An index of—R. Hutchison and H. S. Collier [Eds.], 343.
Treves, Sir F.—Surgical applied anatomy, 170.
Tropical diseases—Sir P. Manson, 171.
Tropics, Fevers in the—Dr. L. Rogers, 257.
Tuberculosis, Diets in — Drs. N. D. Bardswell and J. E. Chapman, 353.
Tuberculosis in infancy and childhood—Dr. T. N. Kelynack [Ed.], 350.
Tuberculosis, Stray leaves and some fruit on cancer and—Dr. H. D. McCulloch, 354.
Tuberculosis, Third annual report of the Henry Phipps Institute for, 259.
Tumours of the cerebellum—Dr. J. W. Wyllie, 262.
Tunstall, Drs. F. J. Warwick and A. C.—First aid to the injured and sick, 274.

Books, Reviews of (*continued*)—
Turner, Dr. W. A.—Epliepsy, 261.
Tweedy and G. T. Wrench, Drs. E. H.—Rotunda practical midwifery, 268.
Urinary surgery, Modern methods of diagnosis in—Dr. E. Deanesly, 272.
Urological surgery, Studies in, 273.
Vagino-peritoneal operations, The technique of—E. Wertheim and T. Micholitsch, 267.
Venins, Les—A. Calmette, 276.
Wallis, F. C.—Surgery of the rectum, 270.
Walsh, Dr. J. J.—History of the medical society of the State of New York, 257.
Warwick and A. C. Tunstall, Drs. F. J.—First aid to the injured and sick, 274.
Wertheim and T. Micholitsch, E—The technique of vagino-peritoneal operations, 267.
Williams, C.—Insanity, its causes and prevention, 263.
Williamson, Dr. R. T.—Diseases of the spinal cord, 259.
Wrench, Dr. G. T.—Rotunda midwifery for nurses and midwives, 268.
Wrench, Drs. E. H. Tweedy and G. T.—Rotunda practical midwifery, 268.
Wyllie, Dr. J.—Tumours of the cerebellum, 262.
Wynter, Dr. W. E.—Minor medicine, 169.
X-ray treatment of skin diseases—Sir M. Morris and S. E. Dore, 351.
Young, Dr. J.—A chronology of medical history, 256.
Brain surgery, Recent progress in, 157.
Breast, Carcinoma of the, 186.
Bristol Medico-Chirurgical Society, 92, 186, 369; Laws of, 370.
Bristol Royal Infirmary, Early history of the — J. P. Bush, 193.
British Medical Association, Sheffield meeting, 280.
Bush, J. P.—Early history of the Bristol Royal Infirmary, 193.

Calmette's ophthalmic reaction, Danger of, 253.

Calmette's ophthalmic reaction in tuberculosis, 154; Dr. J. M. Fortescue-Brickdale, 112.
Carbon monoxide poisoning, 152.
Carcinoma of the bladder, Radical treatment of, 339.
Carter, Dr. T. M.—Dilatation of the posterior cerebral vessels, with basal hemorrhage in a girl aged 16, 322, 375.
Carwardine, T.—Notes on cases, 317; Report on surgery, 246.
Cerebral vessels, Dilatation of the—Dr. T. M. Carter, 322, 375.
Charles, Dr. J. R.—Report on medicine, 61.
Clarke, Dr. J. M.—On the renal changes in a case of hemorrhage into the pons with consequent high blood pressure, 230; Recent work on leukæmia and some allied affections, 289.
Colon, Congenital idiopathic dilatation of the, 244.
Colour vision, Tests for, 252.
Coombs, Dr. C.—Report on medicine, 241; The causation of certain mid-diastolic murmurs, 312.
Cornea, Transplantation of the, 252.

Davies and I. W. Hall, Drs. D. S.—Typhoid fever and typhoid carriers, 39.
Dermatology, Report on—Dr. J. A. Nixon, 160.
Drink problem, The, 85.
Duodenum, Gastro-enterostomy for benign tumours of the, 246.

Ear, Suppurative disease in the nose and—Dr. P. W. Williams, 1.
Eberle, Emily E., Obituary notice of, 376, 379.
Editorial notes, 83, 173, 279, 357.

Feeble-minded, National association for the, Account of Bristol meeting, 382.
Fibroma simulating splenomegaly, Abdominal—Dr. D. A. Alexander, 60.
Filariasis, Case of—A. L. Flemming, 92, 139.
Flemming, A. L.—Case of filariasis with abscess, 92, 139.
Fortescue-Brickdale, Dr. J. M.—Calmette's ophthalmic reaction in tuberculosis, 112.
Fox (Long) lecture on suppurative disease in the nose and ear—Dr. P. W. Williams, 1.
Frontal sinus disease, Osteo-plastic operation for, 189.
Frontal sinus, Suppuration in the, 374.

Gall-stones: in common bile duct, in gall-bladder, Cases of—T. Carwardine, 317.
Gastric ulcer, A study of ten cases of operation for perforated—C. A. Morton, 207.
Gastro-enterostomy for benign conditions of the stomach and duodenum, 246.
Goulden, C. B.—Lachrymal obstruction, its results, dangers and treatment, 143, 187.
Groves, E. W. H.—A case of severe trigeminal neuralgia successfully treated by excision of the Gasserian ganglion, 234; Report on surgery, 65.

Hæmoptysis in pulmonary tuberculosis, Treatment of—Dr. N. Neild, 124.
Hall, Drs. D. S. Davies and I. W.—Typhoid fever and typhoid carriers, 39.
Heart, Alterations in the rhythm of the, 241.
Hemorrhage into the pons with consequent high blood pressure, On the renal changes in a case of—Dr. J. M. Clarke, 230.
Hyperthyroidism, 61.
Hypoparathyroidism, 63.
Hypothyroidism, 62.

Infirmary, Early history of the Bristol Royal—J. P. Bush, 193.
Influenza, The aural complications of—H. F. Mole, 226.
International Congress for Tuberculosis, 86, 358.
Ischæmic paralysis, Volkmann's, 341.

Knight, Herbert Astley, Obituary notice of, 377.

Lachrymal obstruction, On—C. B. Goulden, 143, 187.
Leukæmia and some allied affections, Recent work on—Dr. J. M. Clarke, 289.

Library, Bristol Medical, Bye-laws of, 372.
Library of the Bristol Medico-Chirurgical Society, 89, 183, 286, 366.
Liver, Some complications of ruptured—Dr. L. S. Smith, 238.
Local medical notes, 94, 190, 288, 378.

Medicine, Reports on—Dr. J. R. Charles, 61 ; Dr. C. Coombs, 241 ; Dr. J. M. Fortescue-Brickdale, 335 ; Dr. G. Parker, 152.
Meningeal artery, Rupture of the middle—G. M. Smith, 58.
Meningitis, Basic, with temporary amaurosis, 373.
Mole, H. F.—The aural complications of influenza and their treatment, 226.
Monster, Case of double, 375.
Morton, C. A.—A study of ten cases of operation for perforated gastric ulcer, 207.
Munro, Dr. J. M. H.—Is opsonic treatment useful in phthisis, 97.
Murmurs, The causation of certain mid-diastolic—Dr. C. Coombs, 312.
Muthu, Dr. C.—Some clinical points in the early diagnosis of pulmonary tuberculosis, 131.

National Association for the Feeble-minded, Account of Bristol meeting, 382.
Naval volunteer on duty in Quebec, 1908, A medical—Dr. W. K. Wills, 327.
Neild, Dr. N.—Some notes on the treatment of hæmoptysis in pulmonary tuberculosis, 124.
Neuralgia successfully treated by excision of the Gasserian ganglion, A case of severe trigeminal——E. W. H. Groves, 234.
Nixon, Dr. J. A.—Report on dermatology, 160 ; Surgical aid in chronic ulcer of the stomach, 217.
Nose and ear, Suppurative disease in the—Dr. P. W. Williams, 1.
Nose, Malignant disease of, Two cases of, 93.

Obituary, 376.
Ophthalmia neonatorum, Protargol in the treatment of, 249.
Ophthalmic reaction, Danger of, 253.
Ophthalmic reaction in tuberculosis, 154 ; Dr. J. M. Fortescue-Brickdale, 112.
Ophthalmology, Report on—Dr. C. H. Walker, 249.
Opsonic treatment useful in phthisis, Is—Dr. J. M. H. Munro, 97.

Pancreas, Operative interference in carcinoma of the—Dr. J. Swain, 45.
Pancreatic cyst, Case of—T. Carwardine, 319.
Paralysis of the third nerve, Recurrent, 251.
Parker, Dr. G.—Report on medicine, 152.
Peritonitis, Treatment by drainage of spreading—C. H. Whiteford, 53.
Phthisis, Is opsonic treatment useful in—Dr. J. M. H. Munro, 97.
Polycythæmia, Urobilinuria associated with, 188.
Pons, On the renal changes in a case of hemorrhage into the—Dr. J. M. Clarke, 230.
Pulmonary tuberculosis, The early diagnosis of—Dr. C. Muthu, 131.
Pulmonary tuberculosis, The treatment of hæmoptysis in—Dr. N. Neild, 124.

Renal changes in a case of hemorrhage into the pons—Dr. J. M. Clarke, 230.
Rheumatism and disease of the blood-vessels, 245.
Royal Army Medical Corps, 279.

Scoliosis, Cervical, with hemi-diaphoresis, Case of, 92.
Sick, Preparations for the—
Alexine, 183.
Analgesic balm, 180.
Bornyval, 286.
Bromural, 86.
Carbonic acid baths, 89.
Casona, 366.
Ceridin tablets, 285, 366.
Collargol, 88.
Coryfi 285.
Elixir combreti, 88.
Fermenlactyl, 181.
Formitrol pastilles, 182.
Glycerine extract of red marrow, 182.
Glycerole trypsin, 88.
Glycerophosphates. 86.
Gonosan, 286.

Sick, Preparations for the (*cont'd*)—
Hemisine, 179.
Hémoglobine deschiens, 87.
Holadin, 88.
Junket tablets, 365.
"Ju-Vis" preparations, 182.
Kepler malt extract, 179.
Lactéol tablets, 364.
Lacto-bacteria tablets, 365.
Lecithin, 179.
Lotio pancreatis, 88.
Membroids, 87.
Metramine, 87.
Mostelle, 284.
Novaspirin, 178.
Nutritive elixir of peptones, 182.
Ovaltine, 182.
Palatinoid triple valerianates, 285.
Pill aloin and phenolphthalein compound, 180.
Sauerin tablets, 364.
Soap, Sterilla, 284, 365.
Soloid black mercurial lotion, 88.
Solurol, 181.
Tabloid phenol and menthol compound, 180.
Vaccine, Staphylococcus, 285.
"Vaporole" aromatic ammonia, 285.
Skiagrams, Some fallacies in, 374.
Skin, Tuberculosis of the, 160.
Smith, G. M.—Case of rupture of middle meningeal artery, 58.
Smith, Dr. L. S.—Some complications of ruptured liver, 238.
Society, Bristol Medico-Chirurgical, 92, 186, 369.
Splenomegaly, Abdominal fibroma simulating—Dr. D. A. Alexander, 60.
Stack, E. H. E.—Report on surgery, 157.
Stomach, Gastro-enterostomy for benign conditions of the, 246.
Stomach, Surgical aid in chronic ulcer of the—Dr. J. A. Nixon, 217.
Suppurative disease in the nose and throat—Dr. P. W. Williams, 1.
Surgery, Reports on—T. Carwardine, 246; E. W. H. Groves, 65; E. H. E. Stack, 157; J. Swain, 339.
Swain, Dr. J.—Operative interference in carcinoma of the pancreas, 45; Report on surgery, 339.

Territorial medical service, 95, 177, 380.
Thyroid, Secretory nerves of the, 63.
Tuberculosis in infancy and childhood—Dr. T. N. Kelynack [Ed.], 350.
Tuberculosis, International Congress of, 86, 358.
Tuberculosis of the skin, 160.
Tuberculosis, Ophthalmic reaction in, 154; Dr. J. M. Fortescue-Brickdale, 112.
Typhoid fever and typhoid carriers—Drs. D. S. Davies and I. W. Hall, 39.

Ulcer of the stomach, Surgical aid in chronic—Dr. J. A. Nixon, 217.
Ulcer, Ten cases of operation for perforated gastric—C. A. Morton, 207.
University College, Bristol, Examination successes, 94, 190, 288, 378; Prize distribution, 357.
University College Colston Society, 83, 192.
University for Bristol, 173, 192.
Urobilinuria associated with polycythæmia, 188.

Vascular anastomosis, 66.
Volkmann's ischæmic paralysis, 341.

Walker, Dr. C. H.—Report on ophthalmology, 249.
Whiteford, C. H.—The treatment of spreading peritonitis by drainage, &c., 53.
Williams, Dr. P. W.—The Long Fox lecture on suppurative disease in the nose and ear, 1.
Wills, Dr. W. K.—A medical naval volunteer on duty at Quebec, 1908, 327.

X-ray plates, Ilford, 355.

J. W. Arrowsmith, Printer, Quay Street, Bristol.

### PUBLICATIONS RECEIVED.

From Baillière, Tindall & Cox, London :
*Legal Responsibility of the Drunkard.* By H. Norman Barnett. 1908. Price 2s. 6d. net.
*Mental Deficiency (Amentia).* By A. F. Tredgold. 1908. Price 10s. 6d. net.
*Tuberculosis in Infancy and Childhood.* By Various Writers. Edited by T. N. Kelynack, M.D. 1908. Price 12s. 6d. net.
*Physiological Principles in Treatment.* By W. Langdon Brown, M.D., F.R.C.P. 1908. Price 5s. net.
*Handbook for Attendants on the Insane.* Fifth Edition, revised and enlarged. Published by the Medico-Psychological Association. 1908. Price 2s. 6d. net.
*Operations on the Ear.* By B. Heine. Translated and Edited from the Second German Edition by W. Lombard Murphy, M.A., M.B., B.C. 1908. Price 8s. 6d. net.

From Cassell and Company, Limited, London :
*Estimation of the Renal Function in Urinary Surgery.* By J. W. Thomson Walker, M.B., C.M. 1908.
*Diseases of the Skin.* By Sir Malcolm Morris, K.C.V.O. New and Enlarged Edition. 1908.

From Henry Frowde and Hodder and Stoughton, London :
*The Pathology of the Eye.* By J. Herbert Parsons, B.S., D.Sc., M.D. Volumes I.—III. 1904—1907.
*Diseases of the Eye.* By M. Stephen Mayou, F.R.C.S. 1908. Price 5s.
*The Rectum : its Diseases and Developmental Defects.* By Charles B. Ball, M.Ch. 1908. Price 30s. net.
*Diseases of the Heart.* By James Mackenzie, M.D., M.R.C.P. 1908. Price 25s. net.
*A System of Diet and Dietetics.* Edited by G. A. Sutherland, M.D., F.R.C.P. 1908. Price 30s. net.
*A System of Syphilis.* Edited by D'Arcy Power, M.B., F.R.C.S., and J. Keogh Murphy, M.D., M.C., F.R.C.S. Volume II. 1908. Price 42s. net.

From William Green and Sons, Edinburgh :
*Green's Encyclopedia and Dictionary of Medicine and Surgery.* Volume IX.—*Rhinoliths to Thermotaxis.* [1908.]
*Diet in Infancy.* By A. Dingwall-Fordyce, M.D. 1908. Price 3s. 6d. net.

From Henry Kimpton, London :
*Neurasthenia.* By Gilbert Ballet. Translated by P. Campbell Smith, M.D. 1908. Price 6s. net.

From H. K. Lewis, London :
*The Extra Pharmacopœia of Martindale and Westcott.* Revised by W. Harrison Martindale, Ph.D., and W. Wynn Westcott, M.B. Thirteenth Edition. 1908. Price 10s. 6d. net.
*Clinical Bacteriology and Hæmatology.* By W. D'Este Emery, M.D. Third Edition. 1908. Price 7s. 6d. net.
*The Sexual Disabilities of Man and their Treatment.* By Arthur Cooper. 1908. Price 4s. net.
*Landmarks and Surface Markings of the Human Body.* By Louis Bathe Rawling, M.B., B.C. Third Edition. 1908. Price 5s. net.
*A Manual of Infectious Diseases.* By E. W. Goodall, M.D., and J. W. Washbourn, C.M.G., M.D. Second Edition, revised by E. W. Goodall. 1908.
*Vaccine Therapy and the Opsonic Method of Treatment.* By R. W. Allen, M.D., B.S. Second Edition. 1908.

From Longmans, Green and Co., London :
*Quain's Elements of Anatomy.* Eleventh Edition. Volume III.—*Neurology.* By E. A. Schäfer and J. Symington. 1908. Price 15s. net.

From James Maclehose and Sons, Glasgow :
*A Practical Course of General Physiology.* By D. Noël Paton, M.D., and G. Herbert Clark, M.B., D.P.H. 1908. Price 1s. net.

From Macmillan and Co., Limited, London :
*A System of Medicine.* By Many Writers. Edited by Sir Clifford Allbutt, K.C.B., and Humphry Davy Rolleston, M.A., M.D. Volume IV.—Part I. 1908. Price 25s. net. Volume IV.—Part II. 1908. Price 25s. net.

From "The Medical Review," London :
*An Analytical Index of Volumes I. to X. of "The Medical Review,"* 1898—1907. 1908. Price 7s. 6d. net.

From James Nisbet & Co., Limited :
*Common Affections of the Liver.* By W. Hale White, M.D. 1908. Price 4s. 6d. net.

From Rebman, Limited, London :
*Death and its Verification.* By J. Brindley James. 1908. Price 1s. net.

From The Scientific Press, Limited, London :
*The Care and Nursing of the Insane.* By Percy J. Baily, M.B., C.M. [1908.] Price 2s. 6d. net.

From Snow and Farnham Company, Providence (R.I.) :
*Has Surgical Treatment Lessened the Mortality from Appendicitis?* By Frank P. Vale, M.D. 1908.

From John Wright and Sons, Limited, Bristol :
*A Synopsis of Surgery.* By Ernest W. Hey Groves, M.S., M.D. 1908. Price 7s. 6d. net.
*Natural Therapy.* By Thomas D. Luke, M.D. 1908. Price 7s. 6d. net.

